# Dogs Cannot Bark: Event-Related Brain Responses to True and False Negated Statements as Indicators of Higher-Order Conscious Processing

**DOI:** 10.1371/journal.pone.0025574

**Published:** 2011-10-11

**Authors:** Cornelia Herbert, Andrea Kübler

**Affiliations:** Department of Psychology, University of Würzburg, Würzburg, Germany; University of British Columbia, Canada

## Abstract

The present study investigated event-related brain potentials elicited by true and false negated statements to evaluate if discrimination of the truth value of negated information relies on conscious processing and requires higher-order cognitive processing in healthy subjects across different levels of stimulus complexity. The stimulus material consisted of true and false negated sentences (sentence level) and prime-target expressions (word level). Stimuli were presented acoustically and no overt behavioral response of the participants was required. Event-related brain potentials to target words preceded by true and false negated expressions were analyzed both within group and at the single subject level. Across the different processing conditions (word pairs and sentences), target words elicited a frontal negativity and a late positivity in the time window from 600–1000 msec post target word onset. Amplitudes of both brain potentials varied as a function of the truth value of the negated expressions. Results were confirmed at the single-subject level. In sum, our results support recent suggestions according to which evaluation of the truth value of a negated expression is a time- and cognitively demanding process that cannot be solved automatically, and thus requires conscious processing. Our paradigm provides insight into higher-order processing related to language comprehension and reasoning in healthy subjects. Future studies are needed to evaluate if our paradigm also proves sensitive for the detection of consciousness in non-responsive patients.

## Introduction

The question of what constitutes consciousness has fascinated researchers from different research disciplines for years and centuries. Although yet no clear consensus has been reached most theoretical positions agree with the notion that some phenomena like self-awareness (a sense of self) and specifically higher-order cognitive functions like reasoning and language comprehension (e.g., understanding the meaning of complex text messages) are closely related to consciousness and conscious processing. Recently, several studies investigated the neural correlates of conscious and unconscious stimulus processing in healthy subjects and patients diagnosed with disorders of consciousness. Disorders of consciousness (DOC) (i.e., coma, unresponsive wakefulness syndrome (UWS), formerly vegetative state (VS) [1], and minimally conscious state (MCS) are challenging neurological conditions in which arousal or awareness, or both and thus, consciousness are severely compromised due to focal or diffuse brain lesions following severe head trauma, intracranial haemorrhage or nontraumatic anoxic brain injuries [2]. Using electroencephalographic recordings (EEG) several studies demonstrated that a number of patients diagnosed with DOC respond to simple stimuli and also to more complex semantic stimuli in a similar manner as healthy controls [3], [4], [5], [6]. For example, Kotchoubey et al. [3] investigated event-related brain potentials in a sample of 98 patients with extremely severe and diffuse brain injuries. Fifty patients were diagnosed as being in persistent vegetative state. Cortical processing was investigated with a set of paradigms addressing different levels of information processing. Stimulation included presentation of simple tones, harmonic chords, natural sounds and meaningful, semantic stimuli that were either semantically closely related (e.g., *table-chair*, *I drink tea with sugar*) or semantically unrelated (e.g., *fish-table*, *I drink tea with shoes*). Primary undifferentiated auditory cortical responses expressed in the event-related components P1, N1, and P2 were present in nearly all of the patients. More complex processing related to auditory discrimination (MMN) and stimulus updating (P3) were found in about one-half or one-third of the patients, and evidence for semantic processing as reflected by the N400 was evident in about one-fourth of the patient group. Likewise, several EEG and functional magnetic resonance imaging (fMRI) studies with DOC patients reported enhanced cortical responses to acoustic presentations of the participants' own name (SON) [7]–[12]. In some of these studies, brain activation patterns to the SON in patients with DOC were not differentiable from those observed in healthy controls or patients with locked-in syndrome (LIS), who by definition should be consciously aware, even though in LIS communication might become impossible due to complete muscle paralysis and loss of voluntary control of muscular activity [13]–[15].

However, the question is whether these paradigms really measure higher-order cognitive and conscious processing. Cortical responses to semantically related and unrelated statements could be explained by semantic priming, which in healthy subjects also occurs when primes are presented subliminally [16]. Similarly the SON constitutes a highly overlearned, emotional, personally relevant and familiar stimulus. It is thus processed with a particular high priority and captures the listener's attention quite automatically and effortlessly [17]–[18].

Thus, there is a need for paradigms that allow us to draw clear inferences about conscious-level processing in healthy participants. If such paradigms and the neural responses they evoke had the potential to differentiate effects related to automatic processing and to higher-order, conscious processing they could be also relevant for research in DOC [19]. To contribute to this endeavor, we suggest a new paradigm for the detection of higher-order cognitive processing. The paradigm uses true and false negated sentences (e.g., *dogs cannot bark/fly*) as well as less complex true and false negated prime-target expressions (e.g., *no summer – sun/winter*) and event-related potentials from the EEG as outcome measures. The rationale behind this paradigm is that the truth value of a negated expression can only be correctly evaluated if the meaning of the negated expression is understood. During processing of sentences like *dogs cannot bark/fly*, the words *dog* and *bark/fly* are semantically related or unrelated. However, only in the first case (false negated expressions), the meaning of the expression constitutes a violation of the reader's expectancies from everyday factual knowledge. This violation from peoples' factual knowledge by false compared to true negated expressions can only be correctly detected and evaluated by the individual, if he or she is able to understand and comprehend the meaning of the negated expressions. As outlined in detail below, previous research in healthy subjects already provided some evidence that evaluation of negated language content requires conscious processing. Therefore, it is of particular interest to investigate the neural correlates that are associated with conscious negation processing.

Research into the processing of negations has a long tradition. Seminal research on this topic dates back to the 1970 [20]–[22]. So far, negation effects have most frequently been explored in verification paradigms using true and false un-negated (affirmative) and negated expressions (for an overview see [23]). Most of these behavioral studies corroborated enhanced processing and reaction times for negated compared to un-negated sentences in healthy adults. In addition, in a functional imaging study larger activity increases in the temporal and frontal cortex for negated compared to un-negated sentences were reported [24]. Together these results suggest that processing of negated information is more difficult compared to un-negated content.

EEG-ERP studies investigated the temporal dynamics and cortical correlates underlying the evaluation of true and false negated information. Fischler et al. [25] asked healthy participants to read true and false negated expressions like *a robin is not a tree/bird*. Participants had only little time, i.e., less than a second to evaluate the sentences for their truth value. During reading of false and true sentences, modulation of the N400 potential to the final word (*tree/bird*) showed the opposite pattern of what was implied logically by the negation: N400 amplitudes were larger for *tree* than for *bird*. The N400 potential is sensitive to semantic violations induced either by the stimuli's semantic relatedness within a word or sentence context [26] or by constraints based on subjects' expectations on sentence content [27]. Usually, larger N400 amplitudes are elicited by semantically unrelated (e.g., robin – tree) compared to semantically related (e.g., robin – bird) stimuli. The results reported by Fischler et al. [25] thus imply that during processing of negated statements, the negation is not, at least not initially, integrated into the semantic context. This is in line with two-step models of negation processing that assume that comprehension of negated expressions relies upon the active construal of two mental simulations [28]–[29]. During reading of a negated sentence like “*The door is not open*” the participant initially processes the affirmative core (an open door) and then reverses the polarity of the representation to accommodate the negation (closed door). According to this model, evaluation of the truth value of a negated expression is possible only at the point of time during processing at which the negation is correctly incorporated into the sentence context. In line with this model are results of a second EEG-ERP study which investigated ERP effects for true and false negated statements in a sentence-picture verification task [30]. Participants had either very little time or more than a second to evaluate the meaning of the negated sentence before the verifying target picture was presented. At very short delays (i.e., 300 msec), N400 amplitudes were larger for the semantically incongruent, but with regards to the negation contextually true target words, replicating results from Fischler et al. [25]. In contrast, when participants had more than a second (i.e., 1500 msec) to process the negated expression, N400 amplitudes were modulated in line with the truth value of the negated expression: That is, N400 amplitudes were enhanced for semantically related, but with regards to the negation contextually false target words and reduced for semantically un-related, but with regards to the negation contextually true target words. Similar results were reported by Ferguson et al. [31], who used eye-tracking and ERP methods to delineate the time-course of negation processing. Besides the N400, also later ERPs appeared sensitive to negation processing. Especially the P600, related to the re-integration of semantic anomalies [32]–[33] and the late positive potential (LPP), related to more elaborate stimulus processing and encoding [34] seem to indicate successful integration of the negation into the stimulus context [30]–[31]. Thus, negation processing is not only dependent on semantic comprehension, but also on memory processes and the availability of attentional and cognitive resources, i.e., processes that are thought to require consciousness [35].

Building upon these previous findings, experimental approaches examining specifically those neural correlates that map processes underlying the evaluation of false and true negated stimuli should yield reliable signatures or markers of conscious processing in healthy individuals and possibly in patients with DOC. Past research including the existing three EEG-ERP studies on negation processing mainly used visual stimulus material or a combination of auditory and visual stimulations. Therefore, it is unclear, if in healthy subjects the above reported negation effects also hold for purely verbal and auditory stimulation paradigms. Furthermore, it is unclear if negation-related evaluation effects are the same when assessed at different levels of stimulus complexity (sentence vs. word level). Since up to now, no study exists that investigated these questions in samples of healthy subjects and because vision is often greatly reduced or impaired in DOC, the primary aim of the present study reported here was to investigate the neural correlates of negation processing in healthy subjects for purely auditory stimulation procedures based on language content. To explore the potential of our paradigm for later use in DOC the following open questions were addressed: (1) Which brain potentials vary as a function of the truth value of negated language content when healthy individuals are provided with sufficient processing time to reflect and evaluate the meaning of the presented material and integrate the negation into the semantic context? (2) Are these brain potentials reliable indicators of higher-order cognitive and conscious processing? Stimulus-driven modulations of brain potentials are merely short-lived and elicited within the first 100–200 msec after stimulus presentation. Therefore, we were particularly interested in the modulation of later event-related brain potentials that are sensitive to contextual violations and stimulus evaluation. If modulations of these later ERPs map conscious processes required for the truth value evaluation of a negated expression their amplitudes should reliable differentiate between false and true negated expressions. Accordingly, we expected amplitudes of late ERP potentials to be enhanced for target words related to false negated expressions compared to target words related to true negated expressions. On the other hand, if ERP effects were simply modulated by the semantic relatedness of the words, one would expect the opposite pattern, i.e., larger ERP amplitudes to true relative to false negated expressions. Thus, only in the first case would ERP patterns provide valuable indicators of higher-order cognitive processing functions related to the comprehension of negated language content. To explore the stability of the ERP patterns we report both group and single-subject data.

## Materials and Methods

### Ethics statement

The experiment was conducted in accordance with the Declaration of Helsinki (World Medical Association) and the study was approved by the Institutional Review Board of the Medical Faculty of the University of Würzburg (http://www.ethik-kommission.medizin.uni-wuerzburg.de). All participants gave written informed consent prior to participation.

### Participants

Eighteen healthy adults (14 females, mean age: 25.5 years, SD = 5.11 years), native speakers of German, participated in the study. None of the participants reported any history of chronic somatic, neurological, or psychiatric diseases, or medication use for any of these diseases. Participants had comparable social background, scored normally on questionnaires for mood (Beck Depression Inventory: *M* = 7.36; *SD* = 6.635), state and trait anxiety (Spielberger State Trait Anxiety Inventory: *M* = 36.4; *SD* = 9.1; *M* = 34.9; *SD* = 11.8) and reported normal hearing and normal or corrected to normal vision. Participants were financially reimbursed or received course credits for participation.

### Stimulus Material

Experimental stimuli consisted of negated sentences and negated prime-target pairs that could be true and semantically incongruent or false and semantically congruent. Prime-target expressions consisted of 30 true, semantically incongruent and 30 false, semantically congruent word pairs (e.g., *no summer-winter*, *no summer-sun* etc.). Negated sentences consisted of 32 true, semantically incongruent and 32 false, semantically congruent statements. In these sentences, the negation word appeared directly before the target word, i.e., the word that decided if the sentence was true or false (e.g., *dogs cannot speak*, *dogs cannot bark*, etc.). All stimuli were spoken by a female voice and intonated in standard German. Sentences and prime-target pairs were based on basic factual knowledge and rated for their truth value and congruency by N = 39 healthy adults (28 females) with ages (mean age: 26.8 years, SD = 6.35 years) comparable to the participants of the present study. For all stimulus types the truth value was correctly evaluated. True negated sentences were judged as true and false sentences as false. Likewise, true negated prime-target pairs (e.g., *no summer- winter*) were rated as more congruent compared to false negated prime-target pairs (e.g., *no summer – sun*).

### Experimental Design and Procedure

Prime-target pairs and sentences were presented in separate runs. In each run, true and false statements were presented randomly. The inter-stimulus interval between prime-target expressions was kept constant at 1000 msec (corresponding to a SOA of about 2300 msec). For the sentences, the inter-stimulus interval between the negation word and the target word was 1500 msec (SOA = 1810 msec). Thus, individuals had enough time to process and comprehend the meaning of the presented stimuli before the target word was presented. Sentences and word pairs were separated by an inter-stimulus interval of 2500 msec. Stimuli were presented via stereo loudspeakers. Loudspeakers were placed approximately 90 cm away from the participants left and right ear. During the stimulus presentations, a sound symbol was presented at the centre of a monitor screen placed approximately 80 cm in front of the participants' eyes and participants were asked to look at the symbol throughout the presentation to avoid eye movements during listening. Order of runs was counterbalanced across participants. Before each experimental run, participants were provided with detailed instructions. In the sentence condition participants were told that they would hear a series of sentences that they should evaluate for their truth value. In the word pair condition participants were told that they should listen to the stimuli and evaluate if the meaning of the target word was congruent or incongruent with the negated prime word. They were told to evaluate the stimulus events silently without giving an overt response. After the last run, participants were debriefed in detail about the purpose of the study.

### Physiological data collection and reduction

#### Electroencephalographic recordings

The electroencephalogram was recorded from 28 electrodes with an actiCap system (Brain Products GmBH, Germany). For all electrodes impedance was kept below 10 kOhm. Raw EEG data was recorded continuously at a sampling rate of 500 Hz; FCz served as reference. Off-line, raw EEG signals were digitally re-referenced to an average reference, filtered from 0.01 to 30 Hz and corrected for eye-movement artifacts [36]. In addition, signals exceeding 150 µV in amplitude and below 0.0032 µV and voltage differences greater than 50 µV between two consecutive sampling points were rejected from further analysis. Artifact-free EEG data were segmented separately for the prime-target and the sentence conditions from 500 msec before until 1500 msec after onset of the target word. The 100 msec interval before onset of the target word was used for baseline correction.

Time windows for ERP amplitude scoring were determined for each processing condition (sentences and word pairs) by means of global field power (GFP, [37]). GFP revealed major differences in cortical activity to false and true targets in two consecutive time windows starting from about 300–600 msec and from about 600–1000 msec after onset of the target word. These time intervals were used for comparison of ERPs to true and false targets, both for analysis of group and single subject data.

### Statistical data analysis

For each electrode, subject and processing condition, ERP amplitudes were analyzed as the averaged mean activity (µV) in each of the above reported time windows. Effects were then statistically analyzed separately for each processing condition and time interval with repeated measures analysis of variance (ANOVA). ANOVAs contained each, the factors *category* (true vs. false), and *electrode location* (frontal, centro-parietal, temporal and parieto-occiptal) as within-subject factors. Electrodes included into the factor *electrode location* were grouped as follows: frontal (F4, F3, FCz, Fz, FC1, FC2, FC5, FC6), centro-parietal (CP1, CP2, C4, C3, Cz), parietal (P3, P4, Pz), temporal (T7, T8) and parieto-occiptal (PO9, PO10, P8, P7, O1, and O2), respectively. Significant interaction effects of the factors *electrode location×category* were further decomposed separately for each electrode group (frontal, centro-parietal, parietal, temporal, parieto-occipital) by single ANOVAs, containing the factors c*ategory* and the factor *electrode* (electrodes within the respective electrode cluster) as within-subject factors. When the assumption of sphericity was not met, p-values were adapted according to Greenhouse and Geisser [38].

### Single-subject data (600–1000 msec)

To evaluate the stability of the reported effects, particularly of the late ERP potential differences of the frontal negativity and the parietal positivity, these effects were also determined at the single-subject level for the frontal and parietal electrode clusters.

## Results

### Prime-target condition

In the 300–600 msec time window the factor *electrode location* was significant, *F*(23,391) = 4.5, p = .02, but there was no significant main effect of the factor *category F*(1,17) = 0.74, p = .39, and no significant interaction effect between the factors *category* and *electrode location*, *F*(23,391) = 1.5, p = .17.

**Figure 1:**
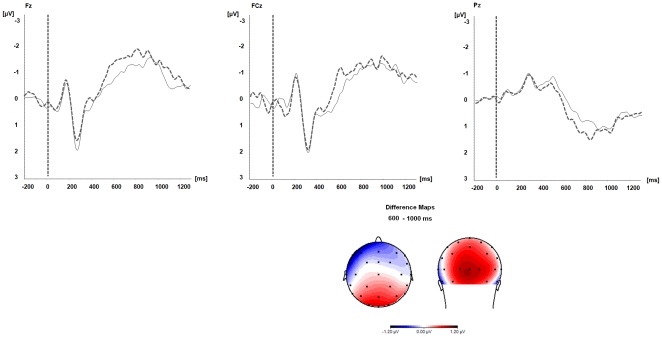
Prime-target condition. Event-related brain potentials elicited during processing of target words preceded by negated prime words. Grey dashed lines: ERPs to false target words. Black lines: ERPs to true target words. Difference Maps: Topographic distribution of the frontal negativity and the LPP. The maps display the difference potentials (µV) of the frontal negativity and the LPP for false compared to true target words.

In the 600–1000 msec time window ANOVAs revealed a significant main effect of the factor *electrode location*, *F*(23,391) = 7.5, p = .0001), and a significant *category×electrode location* interaction, *F*(23,391) = 4.5, p = .001. ANOVAs calculated separately for each electrode group showed that target words preceded by negated, but semantically congruent primes elicited larger cortical negativity at the frontal electrode cluster compared to target words preceded by negated, but semantically incongruent primes, *F*(1,17) = 10.1, p = .006. In the same time window, processing of target words elicited an ongoing cortical positivity at parietal electrodes (P3, Pz, and P4). Amplitudes were again larger for target words preceded by negated primes that were paired with semantically congruent versus incongruent nouns (e.g., *no summer-sun vs. no summer-winter*), *F*(1,17) = 6.7, p = .02. As for the frontal electrode sites, *F*(7,119) = 1.9, p = .14, no significant interaction effect for the factors *category×electrode*, *F*(2,34) = 0.32, p = .61, could be observed. Results are summarized in Figure 1.

### Sentence condition

For targets embedded in a sentence context significant effects were observed in both time windows. In the 300–600 msec time window significant main effects of the factors *electrode location*, *F*(23,391) = 2.8, p = .05, *category*, *F*(1,17) = 9.8, p = .01, and a *category×electrode location* interaction, *F*(23,391) = 4.2, p = .007, were found. ANOVAs calculated separately for each electrode cluster revealed that target words related to false statements elicited significantly larger negative ERP amplitudes at parieto-occipital electrodes compared to targets preceded by true statements, *F*(1,17) = 7.6, p = .01. The category×electrode interaction was not significant, *F*(5,85) = 1.28, p = .29.

**Figure 2:**
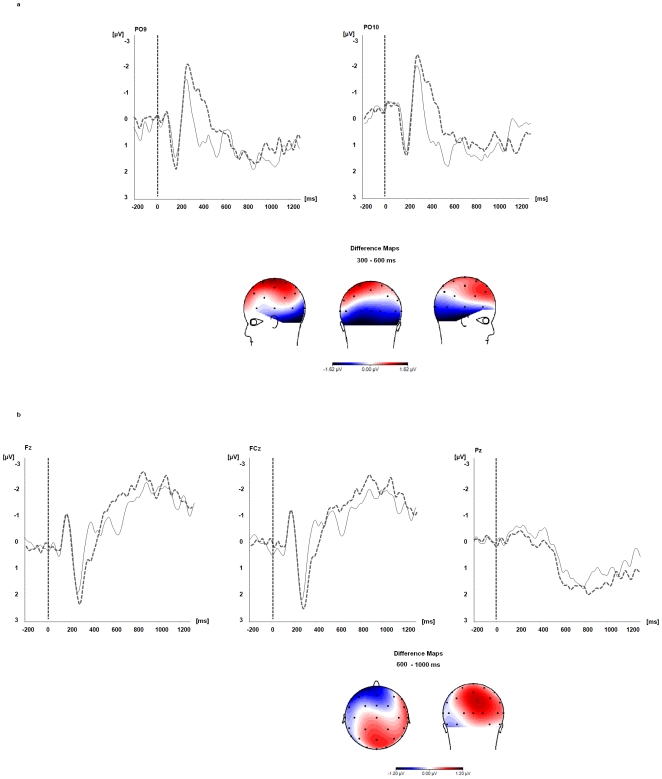
Sentence condition. Event-related brain potentials elicited during processing of target words preceded by true (black lines) and false (grey dashed lines) negated sentence content. Figure 2a: Visual effects. Figure 2b: Frontal negativity and late positive potential (LPP). Difference Maps display difference potentials (µV) for false compared to true target words in the time windows from 300–600 msec (Figure 2a) and from 600–1000 msec (Figure 2b) post target word onset.

In the 600–1000 msec time window significant main effects of *electrode location*, *F*(23,391) = 13.9, p = .001, *category*, *F*(1,17) = 4.9, p = .04, and a significant *category×electrode location* interaction, *F*(23,391) = 2.7, p = .02, were observed. Akin to the prime-target condition, ANOVAs calculated separately for each electrode cluster revealed at frontal electrodes significantly larger negative amplitudes for target words related to false compared to true expressions, *F*(1,17) = 18.4, p = .005, and significantly larger positive amplitudes at parietal electrodes, *F*(1,17) = 17.7, p = .005. Again, no significant *category×electrode* interaction was found, neither for the frontal, *F*(1,119) = 0.9, p = .43, nor for the parietal electrodes, *F*(2,34) = 1.0, p = .36, supporting the stability of the observed ERP patterns within the selected electrode clusters. Results are summarized in Figure 2a and Figure 2b.

### Single subject data (600–1000 msec time window)

Results of individual subjects are summarized in Table 1 and Table 2. Amplitudes of the frontal negativity and the parietal positivity were modulated in the same direction as predicted from the group data when determined for single subjects at the frontal or parietal electrode clusters. For the word pair condition (Table 1) effects could be observed for 83% of the participants (15 of 18 participants) and 89% of the participants (16 of 18 participants) showed larger amplitudes of the frontal negativity and the parietal positivity to false compared to true targets in the sentence condition (Table 2).

**Table 1:** Single subject data (prime-target condition).

Participant	LPP (electrode group)			Frontal negativity (electrode group)		
	true	false	false>true	true	false	false>true
**1**	0.70	2.80	+	0.03	−2.36	**+**
**2**	−0.81	0.26	+	0.77	−1.11	**+**
**3**	0.11	0.23	+	−0.50	−0.93	**+**
**4**	0.87	1.21	+	−0.48	−0.50	**+**
**5**	−0.40	0.36	+	−1.03	−1.05	**+**
**6**	−0.76	−0.42	+	1.78	0.85	**+**
**7**	0.73	0.31	−	−1.55	−1.50	**−**
**8**	0.30	0.47	+	−2.05	−1.71	**−**
**9**	−0.87	0.21	+	0.99	−0.51	**+**
**10**	0.98	2.16	+	−0.18	−0.59	**+**
**11**	2.10	0.65	−	−0.69	−1.05	**+**
**12**	−0.35	1.14	+	−0.29	−0.45	**+**
**13**	−0.46	−0.07	+	0.21	−0.23	**+**
**14**	0.58	2.44	+	−3.05	−2.57	**−**
**15**	0.23	1.47	+	−2.13	−3.11	**+**
**16**	0.05	0.16	+	0.63	0.24	**+**
**17**	0.34	0.89	+	0.49	−1.11	**+**
**18**	0.82	0.79	−	−1.43	−1.48	**+**
**Total (%)**	**3**	**15**	**83%**	**3**	**15**	**83%**

ERP effects (frontal negativity and parietal positivity) of single subjects observed during processing of target words preceded by true or false negated prime words. Columns show amplitude values (in µV) averaged for the frontal or parietal electrode clusters. The + indicates subjects showing larger effects for false compared to true negated statements. The – characterizes subjects showing the opposite effects. Last rows: Total number of subjects (%) showing the pattern false>true.

**Table 2:** Single subject data (sentence condition).

Participant	LPP (electrode group)			Frontal negativity (electrode group)		
	true	false	false>true	true	false	false>true
**1**	1.19	2.47	+	−0.35	−0.42	**+**
**2**	0.29	0.36	+	−0.41	−1.32	**+**
**3**	1.46	2.72	+	−0.69	−2.92	**+**
**4**	0.38	1.73	+	0.51	−0.13	**+**
**5**	0.48	2.31	+	−0.77	−1.40	**+**
**6**	−0.62	−0.20	+	0.19	0.59	**−**
**7**	0.17	0.66	+	−1.48	−1.92	**+**
**8**	1.91	1.77	−	−2.15	−2.49	**+**
**9**	0.09	0.39	+	−0.60	−0.33	**−**
**10**	1.64	2.10	+	−1.93	−3.36	**+**
**11**	0.22	1.28	+	−0.90	−1.92	**+**
**12**	1.13	1.14	+	−0.91	−1.09	**+**
**13**	0.77	2.35	+	−0.14	−1.38	**+**
**14**	1.94	2.55	+	−2.22	−2.80	**+**
**15**	1.11	1.68	+	−2.57	−2.70	**+**
**16**	−0.43	0.30	+	−0.04	−1.11	**+**
**17**	0.69	0.99	+	−0.18	−1.21	**+**
**18**	1.15	0.48	−	−0.49	−1.96	**+**
**Total (%)**	**2**	**16**	**89%**	**2**	**16**	**89%**

ERP effects (frontal negativity and parietal positivity) of single subjects observed during processing of false and true negated sentences. Columns show amplitude values (in µV) averaged for the frontal or parietal electrode clusters. The + indicates subjects showing larger effects for false compared to true negated statements. The – characterizes subjects showing the opposite effects. Last rows: Total number of subjects (%) showing the pattern false>true.

## Discussion

This study examined the neural correlates underlying the evaluation of true and false negated expressions by means of EEG-ERP methods. In contrast to previous research, in the present study a purely verbal and auditory stimulation paradigm was used, no overt response of the participant was required, participants were given enough processing time to mentally evaluate the meaning of the negated expressions and effects of negation were examined both on a word and sentence level. We aimed at finding out if under such processing conditions ERP responses elicited by false and true negated expressions were reliable indicators of higher-order cognitive processing in healthy individuals and could thus be used to determine consciousness and residual cognitive abilities in patients with DOC.

Analysis of ERPs in our sample of healthy individuals revealed that across the word and sentence levels cortical processing was augmented during processing of false target words as compared to true target words. Enhanced cortical processing of false targets was reflected by a frontal negativity potential and an enhanced cortical positivity potential at parietal electrodes, whose amplitudes were both larger for false compared to true target words in the time window from 600–1000 msec post target word onset. Single subject data (see Table 1 and Table 2) confirmed that these ERP patterns are not the result of a few individuals.

Research on language processing suggests that detection of an inconsistency within a semantic context that violates participants' expectancies of common world knowledge is associated with larger amplitudes of the so called N400 potential [27], [39] and accompanied by enhanced amplitudes of a late centro-parietally distributed positivity, the so called P600 potential [27], [32]–[33] or LPP [34]. Whereas modulation of the N400 is assumed to be more directly related with the detection of violations from people's expectancies during language comprehension [26], [40], amplitudes of the P600 or LPP are thought to index memory-based stimulus encoding and post-semantic reintegration processes [34], [41]. Similarly, our findings suggest that detection of an inconsistency within a semantic context that is negated and counterfactual with regard to the listener's expectations is reflected by modulations of a frontal negativity and a P600/LPP like brain potential. Notably, for both conditions (word and sentence conditions), these effects are unlikely to result from automatic priming effects induced by differences between the stimuli's semantic relatedness. In this case, one would have expected larger processing effects for true vs. false target words as true but not false target words were semantically incongruent with the preceding noun. Our results therefore indicate that participants were able to override such automatically activated semantic priming effects and replace them with contextually appropriate contents, which requires that individuals took the meaning of the negation into account.

Theoretically, our results are in good accordance with two factor models of negation processing [23]. These models assume that processing of negation relies on the active construal of two mental simulations including the affirmed and the negated state of affairs. The simulation of these mental models affords considerable processing time before inferences about the truth value of the negated content can be made. Likewise, Deutsch et al. [42] propose that comprehension of negated information is a time- and cognitively demanding process based on intentional and reflective processing. In this view, evaluation of the truth value of a negated expression (even when examined on a word level) cannot be solved automatically, but only by means of higher-order cognitive and conscious processes, a fact that makes the current paradigm very attractive for research on disorders of consciousness.

Previous research into the intricacies of DOC using neurophysiological measures such as event-related brain potentials or functional imaging has focused either on the processing of less complex stimuli, on the processing of personally relevant material or on un-negated, semantically related and unrelated verbal material, whose processing might be explained by more automatic processing. Up to know, very little is known about more complex and conscious cognitive processing in DOC although evidence for conscious processing in DOC is growing. In a recent functional imaging study, Monti et al. [43] investigated 54 patients with disorders of consciousness, 23 patients with unresponsive wakefulness syndrome and 31 patients in a minimally conscious state, with regards to their ability to follow spoken instructions for two mental imagery tasks (tennis playing and spatial navigation). Before each imagery condition verbal cues indicated which imagery condition should be performed. Brain activity elicited during each imagery condition was compared to a resting condition. Both imagery tasks elicit distinct brain activity patterns in the motor cortex (tennis playing) or the parahippocampal gyrus (spatial navigation) in healthy subjects [44]–[45]. Of the 54 patients, 5 patients were able to follow the instructions and wilfully modulate their brain activity in the predicted direction. One patient was able to use the paradigm to answer “yes” or “no” questions during the functional MRI experiment. The results of this multi-subject study are ground-breaking and impressive although the interpretation of the results as evidence for consciousness and wilful action in DOC has been challenged in the literature [46]–[47]. In their reply to Monti, Nachev and Hussein [46], for instance, argue that instructions containing verbal cues like ‘tennis’ or ‘house’ prior to each imagery condition might have been sufficient to automatically elicit brain activation in the respective brain regions of interest simply by means of priming.

By experimental manipulation of the semantic-relatedness of the stimulus material and its truth value our paradigm controls for both: effects attributable to simple priming effects and effects related to language comprehension. Thus, our paradigm could possibly differentiate patients with different levels of consciousness. Regarding patients with DOC we would expect that patients diagnosed with UWS should be unable to evaluate the truth value of a negated expression and respond only to the semantic relatedness of the material. Accordingly, in these patients ERP patterns would point in the opposite direction of what is implied logically by the negation, because true but not false negated expressions contain a semantic violation. MCS patients, in contrast, might respond similar to healthy controls. Together with other tasks and approaches [e.g.], [ 3,19] our paradigm could make an essential contribution to an hierarchical approach to probe the level of consciousness and residual cognitive functions in DOC. In particular, due to the advantage of EEG, our paradigm could be easily used in large patient samples throughout the entire course of the disease without discomfort for the patient. It could be even performed at the patients' home.

Nevertheless, before these goals can be reached, results have to be replicated in larger samples of healthy subjects and different age groups to scrutinize the reliability and validity of the observed effects. To the best of our knowledge, the present study is the first EEG-ERP study to investigate negation processing in the auditory modality for purely verbal material and without requiring an overt behavioral response of the participant. Thus, our EEG-ERP effects are novel, but therefore also only partly comparable with results obtained from previous negation research using predominantly visual material. Specifically, the following issues deserve further investigation: Firstly, in the present study, we found amplitudes of a frontal negativity to vary as a function of the truth value of the negated expression. Previous EEG-ERP negation studies report effects pointing in the same direction as those observed in the current study when comparable delays as those in the current study are used [30]–[31]. However, the effects differ with respect to their topography. In previous EEG studies, processing of true and false negated statements modulated amplitudes of the N400 potential and the N400 had a more centro-parietal distribution. The frontal negativity potential observed in the present study, on the contrary, was most pronounced over frontal and fronto-central electrodes (see Figure 1 and 2b). So far, it is unclear, if these differences can be accounted for by differences in the stimulus material, the sensory modality (visual vs. auditory) or are attributable to different neural processes. Secondly, we found processing of target words related to false negated statements to elicit significantly larger negative ERP amplitudes at parieto-occipital electrodes compared to target words preceded by true negated statements. This effect was significant in the sentence condition and preceded the frontal negativity and LPP effects. Regarding language processing, crossmodal sensory effects are well documented in the literature [48] even in blind people [49]. If functional connectivity between the auditory and the visual modality is preserved in DOC is unclear. Future studies using our paradigm could clarify this point. Thirdly, we compared ERP effects related to true and false negated expressions at very long temporal delays. Given that within a negated context, previous studies demonstrated evaluation of the truth value of an expression to vary as a function of processing time, EEG-ERP studies should also incorporate shorter (possibly also longer) delays between the negated expression and the target stimuli than those used in the present study. This would help to localize the exact time windows during which processing differences between true and false negated expressions can be expected to be most pronounced, particularly when effects of negation are studied with acoustically presented material. Negation is a universal feature of human language and cognition. It is not restricted to factual knowledge. Finally, in future studies, negation could also be used with personally relevant information such as the SON to determine different levels of self-awareness in healthy subjects and in patients with DOC.

In conclusion, our study provides important insight into how true and false negated information is processed. Our study extends the previous negation literature from the visual to the auditory modality and determined the neural correlates underlying the processing and comprehension of negated information. According to our results, paradigms using negated stimulus material could provide valuable insight into higher-order processing related to language comprehension and reasoning in healthy subjects and in patients with DOC, from unresponsive wakefulness to minimal consciousness to full conscious awareness.
